# Compulsory Vaccination for Healthcare Workers in Italy for the Prevention of SARS-CoV-2 Infection

**DOI:** 10.3390/vaccines9090966

**Published:** 2021-08-29

**Authors:** Paola Frati, Raffaele La Russa, Nicola Di Fazio, Zoe Del Fante, Giuseppe Delogu, Vittorio Fineschi

**Affiliations:** 1 Department of Anatomical, Histological, Forensic and Orthopaedical Sciences, Sapienza University of Rome, P. le del Verano 40, 00161 Rome, Italy; paola.frati@uniroma1.it (P.F.); nicola.difazio@uniroma1.it (N.D.F.); zoe.delfante@uniroma1.it (Z.D.F.); delogu.1637299@studenti.uniroma1.it (G.D.); 2 Istituto di Ricovero e Cura a Carattere Scientifico (IRCCS) Neuromed, Via Atinense 18, 86077 Pozzilli, Italy; raffaele.larussa@unifg.it; 3 Department of Clinical and Experimental Medicine, University of Foggia, 71122 Foggia, Italy

**Keywords:** compulsory vaccination, healthcare workers, SARS-CoV-2, Italian legislation

## Abstract

The European Convention on Human Rights (ECHR) judgement no. 116(2021) of 8 April 2021 establishes the principle of mandatory vaccination, indicating the criteria that national legislation must comply with, following the principle of non-interference in the private life of the individual. Vaccination for the prevention of SARS-CoV-2 infection appears to be an essential requirement for providing healthcare assistance. The European experience with compulsory vaccinations, offers a composite panorama, as the strategy of some European countries is to make vaccinations compulsory, including financial penalties for non-compliance. As in other countries, there is a clear need for Italy to impose compulsory vaccination for healthcare workers, in response to a pressing social need to protect individual and public health, and above all as a defense for vulnerable subjects or patients, for whom health workers have a specific position of guarantee and trust. The Italian Republic provided for mandatory vaccinations for health professionals by Decree-Law of 1 April 2021 no. 44, to guarantee public health and adequate safety conditions. As stated by ECHR, the Italian State, despite having initially opted for recommendation as regards to SARS-CoV-2 vaccination, had to adopt the mandatory system to achieve the highest possible degree of vaccination coverage among health professionals to guarantee the safety of treatments and protection of patients’ health. We present the Italian situation on vaccine hesitation in healthcare workers, with updated epidemiological data as well as the doctrinaire, social, and political debate that is raging in Italy and Europe.

## 1. Introduction

The Italian Republic, according to the provisions of Article 32 of its Constitution regarding mandatory treatments, provided for mandatory free vaccinations for health professionals through Law of 28 May 2021 no. 76 (conversion into law, with amendments, of decree-law no. 44 of 1 April 2021, containing urgent measures for the containment of the COVID-19 epidemic, on the subject of SARS-CoV-2 vaccinations, justice and public competitions), to protect public health and to maintain adequate safety conditions in the provision of care and assistance services. Furthermore, vaccination for the prevention of SARS-CoV-2 infection is an essential requirement for practicing the medical profession and for carrying out job performances; the obliged subjects are intended as “healthcare professionals and the workers of healthcare interest”, as highlighted by the Ministry of Health on its institutional website [1]. To be included among the obliged subjects, it is necessary that those belonging to the categories carry out their activity in health, social. or welfare structures, public or private, and in pharmacies, parapharmacies, and professional offices.

Instead, those who carry out services/tasks of different types (e.g., administrative, and commercial workers) in collaboration with or employed by health care professionals and workers of healthcare interest are excluded.

The obligation will persist until the full implementation of the national strategic plan for COVID-19 vaccinations (Law of 30 December 2020, Article 1, paragraph 457), and it will be valid in any case until the end of the current year, except for those for whom a health hazard is ascertained and documented in relation to specific physical conditions.

The need for the introduction of mandatory vaccination derives from the refusal to adhere to the vaccination campaign by a minority of doctors and nurses, thus not guaranteeing the protection of patients’ health [2,3,4]. A recent survey, which was conducted by FADOI (Federation of Associations of Internist Hospital Managers) highlighted different reasons for such refusal [5]. Personal choice was the reason given by only about 1 in 10 healthcare workers (HCWs) who are not vaccinated, with the rest giving several other reasons such as administrative difficulties. About 80% of them stated that the vaccination choice was motivated by the desire to protect themselves as well as other individuals (patients in the first place) [5]. From a recent systematic review dedicated to vaccination hesitation in HCWs, we know that nine studies mentioned that the most common concern about vaccination among HCWs was vaccine safety [6]. Safety concerns mainly included potential side effects, especially long-term ones. Lacking knowledge and appropriate information on vaccine safety, HCWs were reluctant to get vaccinated. They would rather wait for more data to be reviewed, see how the vaccine affects others, and want more information on the safety and efficacy of vaccines [6].

This communication paper aims to present the Italian situation regarding vaccination hesitation in HCWs, with updated epidemiological data as well as the doctrinaire, social, and political debate that is raging in Italy, on a par with what is happening in Europe and worldwide [7,8], at this critical time.

## 2. Judgment 116/2021 of 8 April 2021 of the ECHR

The ECHR judgement no. 116(2021) of 8 April 2021, published a few days after the Italian law promulgation, establishes the general principle of mandatory vaccination, referencing schoolchildren. The Italian provisions faithfully reflect the judgment recently made by ECHR regarding mandatory vaccination, even if the latter concerns schoolchildren.

It recalls the need for vaccination obligatoriness in certain cases, aiming at protecting public health, thus recognizing mandatory vaccination as “necessary in a democratic society” [9,10]. The pronouncement indicates criteria that national legislation must comply with, following the principle of non-interference in an individual’s private life (Article 8 of the ECHR). By means of Article 8, in fact, a public authority cannot interfere with anyone exercising his or her right to private and family life, home, and correspondence. Such interference can only be admitted if provided by the law, when it constitutes a necessary measure, in a democratic society, for national and public security, the economic well-being of the country, defense of order and prevention of crimes, protection of other people health, rights, and freedom [11].

In a historical moment in which vaccination plays a primary role in the prevention of SARS-Cov-2 infection and disease, this ruling constitutes a guide for any legislator, to guarantee the balance between individual rights and protection of public health [12,13].

For the ECHR, the requirements for mandatory vaccination are as follows: (a) means of the law; (b) objectives of protecting individual health, rights, and freedom; and (c) democratic needs.

As regards the first requirement, the sentence specifies that the law must be easily accessible and formulated with sufficient precision, allowing its receivers to regulate their conduct and to foresee the consequences that a given action may entail.

The Court also specified that the term “law” in ECHR Arts. 8 and 11 must be understood in a “substantial” and not “formal” sense. Compulsory vaccination can therefore be provided not only by primary law but also by lower-ranking legal acts.

According to the second requirement, for the European Court, the objective of the legislation is to protect citizens against diseases that can represent a serious risk to health. This refers both to those who receive these vaccinations and to those who cannot be vaccinated and are therefore in a state of vulnerability, hence the importance and the need to achieve a high rate of vaccination within society to protect the weakest. This objective corresponds to the aim of health and rights protection. As regards the third requirement, the Court’s arguments are even more detailed.

Each national authority has the task of evaluating the right balance between public interest and interference in private life, adopting the most suitable means: the greater the importance of individual rights, the lower the State’s margin of appreciation, while whenever interference affects less crucial rights, the State will also have greater possibilities for maneuver [1,14,15]. The ECHR has only a subsidiary role, not being able to assess the needs of the local population and their living conditions as individual states; then, it exercises a final check on the need for interference in individual cases.

In Italy, there is a right of freedom of treatment that can only be overcome by law. HCWs’ obligation derives from the observance of any safety measure provided by science and is confirmed by the obligation to protect colleagues and patients from any risk arising from vaccine refusal.

In any event, the existence of valid reasons preventing vaccination must always be assessed and vaccination can never be considered necessary in the presence of personal contraindications that are justified on a medical and scientific level.

In the constant orientation of the Court, health policy issues leave room for the discretion of national legislator who should assess the balance between objectives to be achieved, available resources, and social needs [16] (Hristozov and others v. Bulgaria (nos. 47039/11 and 358/12, § 119, ECHR 2012).

The Court notes how the recent trend of member countries is to adopt increasingly prescriptive approaches due to a decrease in voluntary vaccination and consequently more difficult in achieving herd immunity. It is not only fundamental to combat no-vax orientations but also to protect the health of all members of society, especially those who are particularly vulnerable to certain diseases and for which the rest of the population is asked to take minimal risk in the form of vaccination (see in this regard Resolution 1845 (2011) of the Parliamentary Assembly of the Council of Europe).

For ECHR, an interference, such as imposing vaccination on certain categories of people and/or workers, is considered “necessary in a democratic society” and justified when it responds to an “urgent social need”, its reasons are “relevant and sufficient”, and the measures are proportionate to the legitimate aim pursued.

## 3. Conclusions

As previously stated, similar to different countries [17,18], there is a clear need for Italy to impose the obligatoriness of vaccination for health professionals, in response to a pressing social need for the protection of individual and public health, and above all as a defense for vulnerable subjects or patients, for whom HCWs have a specific position of guarantee and trust.

Epidemiological data concerning the Italian situation updated to 11 August 2021 show a distribution of cases and COVID-19 related deaths, stratified by age group and sex in the general population, which is useful to compare with the distribution of cases and COVID-19 related deaths among healthcare workers. In addition, data on infections contracted by individual healthcare workers are significant, especially when compared to vaccination rates (Table 1 and Table 2) [19]. However, interpretation of these data requires a necessary critical comment: the general population’s mortality rate is 15 times higher than the one referring to medical class only when extreme age groups are considered. Instead, if we consider the mortality rate of the population group between the age of 20 years and the age of 80 years, the latter is 5 times higher than the one referring to medical class.

**Figure 1:**
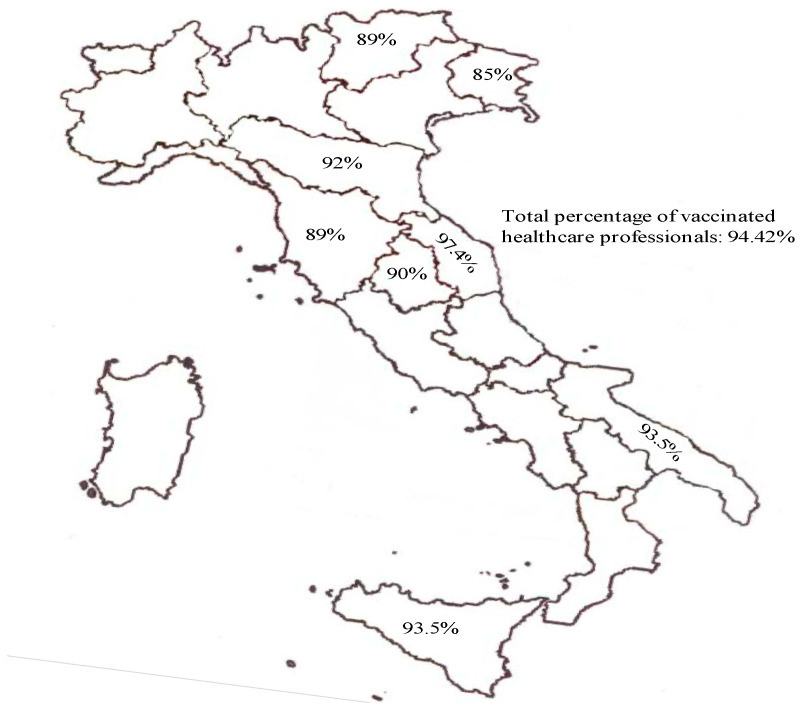
The picture shows single rate of vaccinated healthcare professionals for each region (data communication are incomplete for many regions). Data from Istituto Superiore di Sanità (ISS) are updated to 11 August 2021.

As national data (updated to August 2021), there are 35,691 HCWs without a single dose, or 1.82% of the total. The total number of people is 1,958,461, of whom 94.42% have completed the cycle, while the others are waiting for a recall. These data are compared to 50.86% of the overall population in the age group between 12 and 79 years (considering both those who completed the cycle and those who have only received one dose at the moment) [20,21] (Figure 1 and Table 3).

Considering that vaccination for healthcare workers began much earlier than the campaign for the general population, especially considering the population segment analyzed as demographically homogeneous to the healthcare working-class, particular caution is required in the interpretation of these data. The percentage reached among health workers, although promising, needs further increase by the introduction of a law that establishes its mandatory nature. However, at present, the results achieved may already be considered useful for the prevention of healthcare-related infections.

In Table 4 are summarized the epidemiological data relative to the estimated vaccination efficacy in the Italian population aged >12 years in cases of COVID-19 cases diagnosed in the period 4 April–8 August 2021, with 95% confidence interval.

Moreover, in line with what is stated by ECHR themselves, the Italian State, despite having initially opted for the form of recommendation, subsequently had to adopt a mandatory system to achieve the highest possible degree of vaccination coverage among health professionals to guarantee the safety of the treatments and the protection of the health of the patients. It is always the Court that reiterates that, in matters of health policy, it is up to the national authorities to decide, because they are in the best position to assess their priorities, the use of the resources, and the social needs.

The principle of proportionality, previously mentioned through the judgment no. 116 (2021) of ECHR, is also respected by the Italian legislative provision. The exemption is foreseen only for professionals who have contraindications; then, the mandatory nature is guaranteed by the application of sanctions, and not by the execution of coercive health treatments [22]. Moreover, economic protection systems are guaranteed with the provision of adequate compensations [23,24]. The most controversial point, however, concerns the safety of vaccines.

Equally precise seems to be the reference, to consensus, to the vital importance of the means of protecting populations against diseases that can have serious effects on individual health and which, in the event of serious outbreaks, can cause damage to the entire community in case of serious outbreaks [25,26,27]. The balance between risk and expected benefits is fundamental.

In consideration of the risks, quite rare even in the case of COVID, but sometimes very serious for the individual’s health, the ECHR previsions stressed the importance of taking the necessary precautions before vaccination [28,29], checking each individual for possible contraindications.

There is also a need for constant monitoring of the safety of vaccines in use by the state drug authority: AIFA (Italian Drug Agency), which manages the pharmacovigilance related to the Italian vaccination campaign, periodically publishes bulletins containing adverse reactions, deaths, and investigations on specific vaccine batches; the latest information available, relating to data up to August 2021 [30], stated that the reporting rate of adverse effects on the overall population equals 0.13‰ of which the great majority are of mild intensity and general nature, for all four currently available vaccines (Pfizer/BioNTech, Astra Zeneca, Moderna, Janssen Cilag). With reference to the Vaxzevria vaccine (Astra Zeneca), then, the lack of statistical correlation with age and sex for the adverse event consisting in thromboembolism has allowed its reintegration among the vaccines approved by the EMA (European Medicines Agency), which has judged the benefits deriving from its use as exceeding the risks of adverse effects.

Recently, it was stated that healthcare workers have also identified the too-fast vaccine trials as a reason to be hesitant to vaccinate [31]. There is an urgent need, therefore, for more health-related education among healthcare workers to alleviate any fears associated with the vaccine. Finally, unavoidable needs are those to design effective and evidence-based strategies to promote the COVID-19 vaccine’s acceptance among healthcare workers.

In conclusion, we can state that the legislation on vaccines is generally conditioned, in its essential elements, by two factors: the validity of the results of medical-scientific research, in constant evolution, on the safety and efficacy of vaccines [32]; and the sanitary and epidemiological conditions periodically ascertained by the authorities in charge [33]. In the light of these arguments, the legislator must make a reasonable balance between the need to guarantee effective prevention and that of not over-constraining the right to self-determination of the individual who does not intend to undergo health treatment.

The European experience with compulsory vaccinations, as recently reconstructed by a review, offers a composite panorama, as the strategy of some European countries is to make vaccinations compulsory, including financial penalties for non-compliance [34]. In some countries, childhood vaccinations have been made mandatory in response to declining vaccination rates and outbreaks of vaccine-preventable infectious diseases, including measles [35]. The question of the effectiveness of compulsory vaccination policies remains open, as they are directly influenced by vaccination rates that are conditional on the achievement of the herd immunity threshold [36].

Italy became the first country in Europe to make COVID vaccination mandatory for healthcare workers [37,38]. Obligatoriness or recommendation must always be contextualized, so in general, a high voluntary vaccination coverage tends to be accompanied by a model based on recommendation, while lower and lower coverage can lead to opting for obligatoriness, with the fair warning that “*there is always a small minority of people you will not reach, or whose minds you will not change…As the vaccination program continues, social norms around COVID-19 vaccines will become more entrenched, people will see that their friends, colleagues and loved ones have been vaccinated, and have been well. Levels of hesitation will probably drop*” [8,39,40].

## Figures and Tables

**Table 1:** Distribution of cases (*N* = 4,409,090) and COVID-19 related deaths in Italy (*N* = 127,476) stratified by age group and sex (produced by the Istituto Superiore di Sanità (ISS), Rome. Data updated to 11 August 2021).

Age Group (Years)	Male Sex					Female Sex					Total Cases				
	N. Cases	% of Total Cases	N. Deaths	% of Total Deaths	Lethality %	N. Cases	% of Total Cases	N. Deaths	% of Total Deaths	Lethality %	N. Cases	% of Total Cases	N. Deaths	% of Total Deaths	Lethality %
0–9	125,741	5.8	7	<0.1	<0.1	117,310	5.2	7	<0.1	<0.1	243,051	5.5	14	<0.1	<0.1
10–19	232,996	10.8	9	<0.1	<0.1	213,201	9.5	8	<0.1	<0.1	446,197	10.1	17	<0.1	<0.1
20–29	277,988	12.9	44	0.1	<0.1	264,198	11.8	25	<0.1	<0.1	542,190	12.3	69	0.1	<0.1
30–39	270,541	12.5	163	0.2	0.1	281,633	12.5	98	0.2	<0.1	552,178	12.5	261	0.2	<0.1
40–49	334,569	15.5	795	1.1	0.2	367,660	16.4	340	0.6	0.1	702,230	15.9	1135	0.9	0.2
50–59	370,193	17.1	3274	4.5	0.9	384,764	17.1	1278	2.3	0.3	754,959	17.1	4552	3.6	0.6
60–69	247,693	11.5	9527	13.2	3.8	229,484	10.2	3624	6.5	1.6	477,178	10.8	13,151	10.3	2.8
70–79	175,240	8.1	21,774	30.2	12.4	170,178	7.6	10,403	18.8	6.1	345,418	7.8	32,177	25.2	9.3
80–89	105,239	4.9	27,905	38.7	26.5	151,879	6.8	23,512	42.4	15.5	257,125	5.8	51,417	40.3	20.0
≥90	21,485	1.0	8528	11.8	39.7	66,977	3.0	16,153	29.1	24.1	88,462	2.0	24,681	19.4	27.9
Unknown age	51	0.0	1	0.0	2.0	51	3.0	1	0.0	2.0	102	0.0	2	0.0	2.0
Total	2,161,736	49.0	72,027	56.5	3.3	2,247,335	51.0	55,449	43.5	2.5	4,409,090	-	127,476	-	2.9

Note: table does not include cases where sex is not known.

**Table 2:** Distribution of cases (*N* = 137,082) and healthcare workers COVID-19 related deaths in Italy (*N* = 333) stratified by age group and sex (produced by the Istituto Superiore di Sanità (ISS), Rome. Data updated to 14 July 2021).

Age Group (Years)	Male Sex					Female Sex					Total Cases				
	N. Cases	% of Total Cases	N. Deaths	% of Total Deaths	Lethality %	N. Cases	% of Total Cases	N. Deaths	% of Total Deaths	Lethality %	N. Cases	% of Total Cases	N. Deaths	% of Total Deaths	Lethality %
18–19	5226	12.7	0	0	0	12,418	12.9	0	0	0	17,644	12.9	0	0	0
30–39	8790	21.4	1	0.4	0	17,774	18.5	2	1.9	0	26,564	19.4	3	0.9	0
40–49	8917	21.7	10	4.3	0.1	27,637	28.8	8	7.8	0	36,554	26.7	18	5.4	0
50–59	10,767	26.2	41	17.8	0.4	30,255	31.5	27	26.2	0.1	41,022	29.9	68	20.4	0.2
60–69	6740	16.4	117	50.9	1.7	7350	7.7	29	28.2	0.4	14,090	10.3	146	43.8	1
70–79	466	1.1	35	15.2	7.5	222	0.2	9	8.7	4.1	688	0.5	44	13.2	6.4
Unknown Age	225	0.5	26	11.3	11.6	295	0.3	28	27.2	9.5	520	0.4	54	16.2	10.4
Total	41,131	30	230	69.1	0.6	95,951	70	103	30.9	0.1	137,082	100	333	99.9	0.1

Note: table does not include cases where sex is not known.

**Table 3:** Vaccination coverage in the Italian population aged >12 years and COVID-19 cases diagnosed in the last 30 days, by vaccination status and age group (produced by the Istituto Superiore di Sanità (ISS), Rome. Data updated to 23 July 2021).

Group	Age Group	Unvaccinated People	Single Dose	Complete Cycle
Population (3 July 2021)	12–39	13,017,353 (74.7%)	2,651,558 (15.2%)	1,766,644 (10.1%)
	40–59	8,163,811 (44.3%)	6,157,091 (33.4%)	4,126,558 (22.4%)
	60–79	3,118,561 (23.0%)	4,825,699 (35.6%)	5,628,519 (41.5%)
	80+	446,128 (9.8%)	245,504 (5.4%)	3,862,475 (84.8%)
Diagnosis of SARS-CoV-2 (18 June 2021–18 July 2021)	12–39	19,080 (81.0%)	3313 (14.1%)	1167 (5.0%)
	40–59	5457 (60.2%)	2357 (26.0%)	1256 (13.8%)
	60–79	1446 (43.0%)	1090 (32.4%)	830 (24.7%)
	80+	301 (33.2%)	53 (5.8%)	552(60.9%)

**Table 4:** Estimated vaccination efficacy in the Italian population aged >12 years in cases of COVID-19 cases diagnosed in the period 4 April–8 August 2021, with 95% confidence interval (Produced by the Istituto Superiore di Sanità (ISS), Rome. Data updated to 11 August 2021).

Group	Age	Vaccination Efficacy % (Incomplete vs. Unvaccinated) *	Vaccination Efficacy % (Complete vs. Unvaccinated) *
SARS-2 diagnosis	12–39	41.34 (40.45–42.22)	68.32 (67.6–69.02)
	40–59	66.63 (66.08–67.17)	79.00 (78.59–79.41)
	60–79	75.51 (75.05–75.96)	88.01 (87.71–88.31)
	80+	52.73 (51.2–54.22)	88.91 (88.58–89.23)
	Total	62.06 (61.71–62.41)	82.33 (82.11–82.54)
Hospitalizations	12–39	79.27 (75.63–82.53)	88.44 (85.42–91.00)
	40–59	89.49 (88.32–90.58)	94.00 (93.09–94.82)
	60–79	86.55 (85.8–87.26)	95.53 (95.08–95.94)
	80+	65.38 (63.27–67.38)	94.07 (93.69–94.44)
	Total	82.32 (81.68–82.95)	94.7 (94.44–94.95)
Intensive care admissions	12–39	- **	- **
	40–59	92.09 (88.51–94.82)	97.15 (94.84–98.63)
	60–79	90.57 (88.96–92.01)	97.79 (96.94–98.46)
	80+	74.86 (66.32–81.63)	95.79 (94.22–97.01)
	Total	89.4 (87.93–90.74)	97.16 (96.49–97.74)
Deaths	12–39	- **	- **
	40–59	86.82 (79.33–92.19)	95.13 (89.47–98.26)
	60–79	89.44 (87.98–90.76)	96.89 (95.91–97.7)
	80 *	74.22 (71.74–76.53)	96.69 (96.27–97.07)
	Total	82.26 (80.91–83.53)	96.82 (96.45–97.15)

* Efficacy estimates with 95% confidence interval are shown in the table; ** estimates not calculable due to low frequency of events in some groups.

## Data Availability

Data available at https://www.epicentro.iss.it/coronavirus/bollettino/Bollettino-sorveglianza-integrata-COVID-19_11-agosto-2021.pdf (accessed on 24 August 2021).
